# Development of an accurate classification system of proteins into structured and unstructured regions that uncovers novel structural domains: its application to human transcription factors

**DOI:** 10.1186/1472-6807-9-26

**Published:** 2009-04-30

**Authors:** Satoshi Fukuchi, Keiichi Homma, Yoshiaki Minezaki, Takashi Gojobori, Ken Nishikawa

**Affiliations:** 1 Center for Information Biology & DNA Data Bank of Japan, National Institute of Genetics, Yata 1111, Mishima, Shizuoka 411-8540, Japan; 2 Department of Bioinformatics, Maebashi Institute of Technology, Kamisadori 460-1, Maebashi, Gunma 371-0816, Japan

## Abstract

**Background:**

In addition to structural domains, most eukaryotic proteins possess intrinsically disordered (ID) regions. Although ID regions often play important functional roles, their accurate identification is difficult. As human transcription factors (TFs) constitute a typical group of proteins with long ID regions, we regarded them as a model of all proteins and attempted to accurately classify TFs into structural domains and ID regions. Although an extremely high fraction of ID regions besides DNA binding and/or other domains was detected in human TFs in our previous investigation, 20% of the residues were left unassigned. In this report, we exploit the generally higher sequence divergence in ID regions than in structural regions to completely divide proteins into structural domains and ID regions.

**Results:**

The new dichotomic system first identifies domains of known structures, followed by assignment of structural domains and ID regions with a combination of pre-existing tools and a newly developed program based on sequence divergence, taking un-aligned regions into consideration. The system was found to be highly accurate: its application to a set of proteins with experimentally verified ID regions had an error rate as low as 2%. Application of this system to human TFs (401 proteins) showed that 38% of the residues were in structural domains, while 62% were in ID regions. The preponderance of ID regions makes a sharp contrast to TFs of *Escherichia coli *(229 proteins), in which only 5% fell in ID regions. The method also revealed that 4.0% and 11.8% of the total length in human and *E. coli *TFs, respectively, are comprised of structural domains whose structures have not been determined.

**Conclusion:**

The present system verifies that sequence divergence including information of unaligned regions is a good indicator of ID regions. The system for the first time estimates the complete fractioning of structured/un-structured regions in human TFs, also revealing structural domains without homology to known structures. These predicted novel structural domains are good targets of structural genomics. When applied to other proteins, the system is expected to uncover more novel structural domains.

## Background

Recent studies revealed that a high fraction of proteins in eukaryotes have long stretches of intrinsically disordered (ID) regions [1,2]. Proteins with ID regions, abundant in the cytosol and nucleus but scarce in mitochondria [3], are frequently involved in cellular regulatory processes such as transcription, translation, and cellular signaling transduction [4-7]. The abundance of proteins with ID regions in the cells can be tightly controlled by regulation of transcript clearance, proteolytic degradation, and translational rate[8]. Transcription factors (TFs) such as activators, repressors, or enhancer-biding factors may be considered typical, as most of them contain long stretches of ID regions [9,10]. While human TFs are characterized by a DNA-binding domain (DBD) and other structural domains, 60% of them are composed only of a DBD and ID regions [9]. Intriguingly some ID regions in TFs harbor functional sites called transactivation domains (TADs), which interact with coactivators and other factors of the pre-initiation complex to transmit the activation signal to RNA polymerase. *In vitro *experimental studies, particularly those with NMR spectroscopy, revealed that TADs of various TFs are unstructured in isolation, but become structured upon binding to their interaction partners [4,11-17]. Prokaryotic TFs differ from eukaryotic TFs in that they generally do not have long ID regions; DBD and/or other structural domains occupy nearly the entire lengths[18,19]. The molecular architecture composed of structural domains and ID regions is generally found in eukaryotic proteins, including membrane proteins [20]. Highly sensitive homology search tools, such as PSI-BLAST [21] and HMMER [22], made it possible to identify and locate structural domains along a protein sequence with high confidence [23], if at least one homolog had its 3D structure experimentally determined. According to the general view above, the regions of the protein to which no known structure has been assigned should correspond either to ID regions or to domains of unknown structure, which we hereafter call "cryptic" structural domains. Thus, if ID regions can be accurately distinguished from cryptic domains, the entire length of any protein can be classified into structural domains and ID regions.

Efficient computer programs have been developed for prediction of ID regions from protein sequences [24-27] and utilized in genome-wide surveys [1,7,28]. All of these prediction methods are based on the fact that ID regions have a characteristic skewed amino acid composition; hydrophilic and charged residues are abundant, while hydrophobic residues are scarce [29,30]. In the previous study [9], we employed the profile-based disorder prediction program, DISOPRED2 [3], together with a domain identification method. In 401 human TFs, the residue-wise fractions of structural domains and ID regions were found to be 31% and 49%, respectively, with the remaining 20% left unclassified. As some of the unclassified regions were long, they can possibly contain new structural domains[31]. Others showed mosaic patterns consisting of short ID and unclassified regions.

Besides the skewed amino acid composition, ID regions are characterized by higher sequence divergence as compared to structural domains. It is known that structural domains are well conserved through evolution and can be detected by homology search methods even across different kingdoms (prokaryotes and eukaryotes, for example) [32,33]. Sequence conservation has been used as a factor to discriminate structural domains from domain-linkers [34,35]. By contrast the sequences of ID regions generally mutate more rapidly than structural regions, although some exceptions were found [36]. Presumably because ID regions are not structurally constrained [20], frequent indels (insertions/deletions), un-aligned sites, and amino acid substitutions occur in ID sequences. It is common that BLAST searches using human proteins as queries detect homology in ID regions in mammalian proteins, but in a very small number of invertebrate proteins, and in none of the other more remote species, although homology in structural domains is generally detectable beyond invertebrates.

In the present study, we developed a program to CLAssify DIsorder regions and STructural domains, CLADIST, incorporating information of un-aligned sites into amino acid composition. We developed a combined system that uses structural domain identification, disorder prediction, and the CLADIST program to make order/disorder assignments to the entire length of proteins. We found that our system, DICHOT, divides proteins into structural domains and ID regions highly accurately. Application of the system to human TFs identifies ID regions and structural domains, including a number of cryptic structural domains.

## Results

### Utilization of sequence conservation in structure/disorder classification

**Figure 1:**
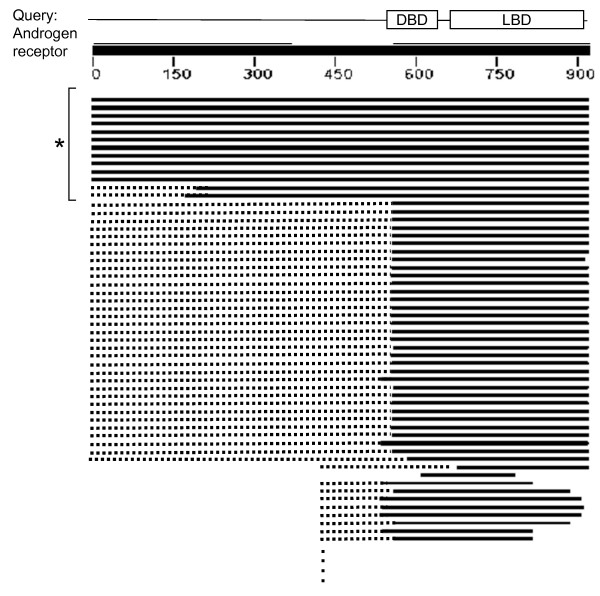
**Sequence alignment pattern of a protein with a long ID region**. At the top, the domain structure, the sequence of the human androgen receptor (hAR), and a residue number scale are presented. Below them, some of the high-scoring homologues found by a BLAST search conducted with hAR as query against Swiss-Prot are presented, where the solid bars represent aligned segments and the dotted ones do un-aligned segments. The star signifies mammalian orthologues: the N-terminal sections of paralogues, such as the progesterone receptor and the glucocorticoid receptor, cannot be aligned to hAR. The bar representation output of the BLAST server  was modified.

Figure 1 presents the human androgen receptor (hAR) as an example of the absence of BLAST alignment in the ID regions. The C-terminal half composed of a DBD and a ligand binding domain (LBD) [37,38] is well conserved. By contrast, the N-terminal half, containing the functional region, AF1, and is mainly composed of ID regions [9,13], is so diverged that the alignments over entire length is possible only among mammalian orthologues (the topmost 13 homologues in the figure). Homology was detected by BLAST only in the C-terminal half even in the human paralogues, the progesterone/glucocorticoid receptors. As this example illustrates, a difference in sequence divergence between structural domains and ID regions exists, which can be exploited in classification.

We thus developed the CLADIST program that uses the amino acid composition considering information of highly divergent sequences in ID regions to divide the entire proteins into structural domains and ID regions. The program treats alignment gaps and un-aligned regions as the 21st element of amino acid composition and uses support vector machine for classification (for details, see Materials and Methods).

We tested the accuracy of the CLADIST program in classifying proteins into structural domains and ID regions. CLADIST was applied to a set of 58 proteins with experimentally verified ID regions selected from the DisProt database [39] (see Materials and Methods). Table 1 shows benchmark tests performed in 4-fold validation test. In test 1, all BLAST homologues with e-value less than 10^-3 ^were used for estimation of the local amino acid composition. 92.4% of the residues were correctly assigned to either structural domains or ID regions. In order to examine the cases in which only close homologues of a query are available, we only took BLAST homologues with e-value less than 10^-100 ^to perform test 2. In this test, the percentage of correctly assigned residues dropped to 86.1% (Table 1). This test simulates cases of lineage-specific proteins, with only closely related homologues whose entire lengths including ID regions are aligned by BLAST. In such cases, sequence divergence cannot be effectively utilized for classification, leaving the local amino acid composition (with few gaps) as the sole factor to rely on. In other words, the factor of sequence divergence with incorporation of alignment gaps increases the accuracy by more than 6%.

**Table 1:** Benchmark tests of the CLADIST program and the DICHOT system.

	A	B	C	D	E
Test1	3546	200	1303	194	92.4%
Test2	3573	173	994	503	86.1%
Test3	3673	73	1448	49	97.7%

A: Number of correctly assigned residues in structural domains

B: Number of incorrectly assigned residues in structural domains

C: Number of correctly assigned residues in ID regions

D: Number of incorrectly assigned residues in ID regions

E: Percentage of correctly assigned residues given by 100 × (A + C)/(A + B + C + D)

Test1: CLADIST under standard conditions (using homologues with e-values less than 10^-3^); Test2: CLADIST using homologues with e-values less than 10^-100^; Test3, The DICHOT system

### The DICHOT system

From the result above, we reasoned that combining accurate structural domain assignment by profile methods, DISOPRED2 prediction, and the CLADIST classification program could lead to a complete assignment of protein molecules into structural domains and ID regions with high accuracy (see Materials and Methods for detail). We built the DICHOT system by giving the first priority to trans-membrane (TM) domains and structural domains assigned by alignments to PDB sequences, then to ID regions predicted by DISOPRED2, and finally to both structural domains and ID regions assigned by CLADIST. In four steps, DICHOT classifies the entire sequence of a query into structural domains and ID regions. Structural domains consist of "known domains", i.e., structural domains with similarity to known 3D structures, and "cryptic domains" signifying structural domains without similarity to known 3D structures.

**Figure 2:**
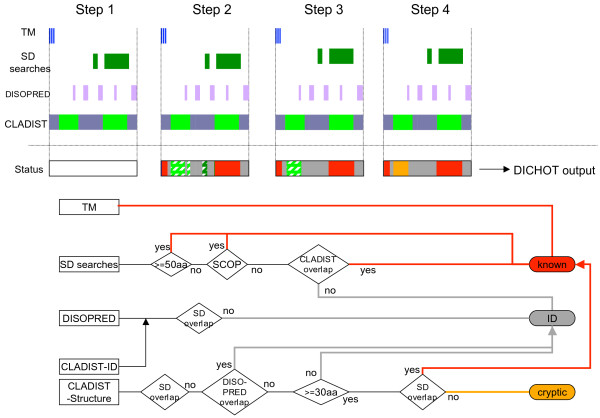
**Schematic illustration of the DICHOT system**. Structural domain and ID region assignments by different methods are presented at the top, the status boxes are displayed in the middle to illustrate the classifications after the corresponding steps, and a flow chart is shown in the lower half. Data processing proceeds from left to right. In the upper-most four rows, results of trans-membrane assignments, structural domain (SD) searches, DISOPRED2 prediction, and CLADIST prediction of a hypothetical query sequence are depicted, with the vertical dotted lines marking the N- and C- termini of the query. The blue, green, and purple bars respectively represent a trans-membrane region, regions structurally aligned by homology searches, and ID regions predicted by DISOPRED2, while the alternating purple and light green segments signify the ID regions and structural domains predicted by CLADIST, respectively. The red and gray bars stand for known domains and ID regions, respectively, while the orange section denotes a cryptic domain.

A flow chart of the DICHOT system is presented in Figure 2 together with region assignment steps of a hypothetical protein, in which the tentative status after each step is shown in the status box. In the first step, a homology search against the PDB sequences (SD search in the figure), trans-membrane (TM) assignments, DISOPRED2 prediction, and CLADIST prediction are carried out. The assignment of known domains is firstly carried out in step 2 (the red bars in the status box). The ID regions predicted by DISOPRED2 and CLADIST are also accepted, when they lie outside of the known domains (the gray bars). DICHOT employs length cutoffs for known and cryptic domains. The hatched boxes in step 2 are regions that fall below the length cutoffs, and are assessed by referring to the SD search and CLADIST results in steps 3 and 4. In this case, the left-most hatched box is classified as a cryptic domain, because it lies outside of any of the known domains, while the other two hatched boxes are not so classified because they are judged unqualified in step 3 (see Materials and Methods for details). Any query sequence is thereby entirely classified into two categories, structural domains consisting of known and cryptic domains, and ID regions (the last status box).

Application of the DICHOT system to the above-mentioned test data showed that the accuracy increases to 97.7% (Test 3 in Table 1). The increase of accuracy can be attributed to the following two factors. First, prediction error in structural domains can be reduced by the accurate domain assignments by the homology searches against PDB, which are not included in CLADIST but in DICHOT. Second, prediction error in ID regions can be reduced by taking the intersection of the ID regions obtained by CLADIST and DISOPRED2. Therefore the combined system was effective in improving the reliability of assignments. If used alone, CLADIST misclassifies some residues in structural domains into ID regions. On the other hand the DICHOT system gives highly accurate structural domain assignments with the employment of profile methods, resulting in the increase in accuracy.

### Application of DICHOT to human transcription factors

**Figure 3:**
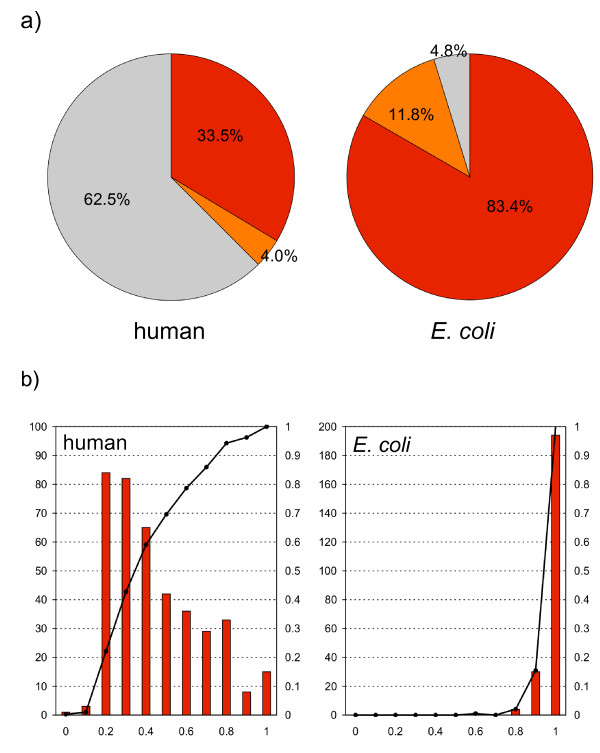
**Fractions of human and *E. coli *TFs occupied by structural domains and ID regions**. a) Overall statistics of structural domains and ID regions in human and *E. coli *TFs. The red, orange, and gray sectors represent the fractions of residues in known structural domains, cryptic domains, and ID regions, respectively. b) Histograms of TFs sorted according to fraction ranges occupied by structural domains and cumulative frequencies. The red bars show the frequency, while the black lines connecting dots represent the cumulative frequencies. The fractions of structural domains are plotted along the x axis. The scale on the left is for the number of TFs, while the right scale is for the cumulative frequency.

The DICHOT system was applied to a set of 401 human TFs to classify each protein into structural domains and ID regions. Out of a total of 219,628 residues, 33.5% were classified as known structural domains, 4.0% as cryptic structural domains, and 62.5% as ID regions (Fig. 3a). Compared to the previous results (31% known structural domains, 49% ID regions, and 20% unassigned), the fractions of structural domains and ID regions both increased significantly. The fractional increase in known structural domains (from 31% to 33.5%) is attributable to the increase in 3D structural data in PDB over the previous study. For comparison, DICHOT was also applied to 229 TFs from *E. coli *(Fig. 3a): 83.4%, 11.8%, and 4.8% of the residues fell on known structural domains, cryptic structural domains, and ID regions, respectively. There is a clear difference between human and *E. coli *TFs: unlike human TFs, *E. coli *TFs consist almost entirely of structural domains, with a small fraction of ID regions corresponding to relatively short linkers connecting structural domains and/or terminal tails.

Figure 3b presents the fractions of structural domains and ID regions in human TFs (upper panel) and *E. coli *TFs (lower panel) in a different format. For example, the tallest bar in the upper panel indicates that between 10 and 20% of the residues in 91 human TFs were in structural domains, or, equivalently between 80 and 90% were in ID regions. The cumulative graph shows that 60% of the human TFs have less than 40% of the residues in structural domains, in other words, have more than 60% in ID regions. In contrast, in most *E. coli *TFs structural domains account for more than 90% of their lengths and long ID regions are absent.

**Figure 4:**
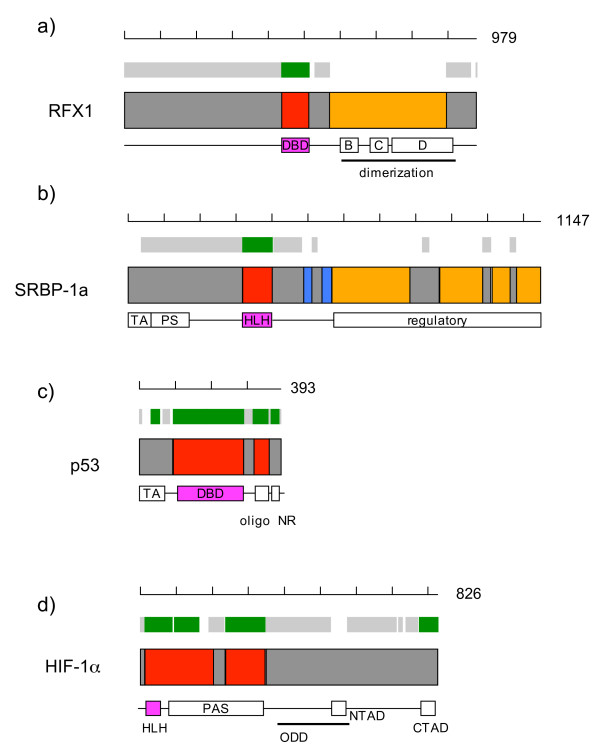
**Examples of structural domain and ID region assignments**. Structural domain and ID region assignments to four human TFs are presented. From top to bottom, each diagram consists of a scale with the total number of amino acid residues, assignments in the previous report, assignments in this study, and domain architecture from the literature. In the previous assignments, structural domains, ID regions, and un-assigned sections are presented in green, gray, and white, respectively. In the present assignments, domains of known structure, cryptic structural domains, and ID regions are respectively colored in red, orange, and dark gray. In the domain architecture derived from the literature, pink boxes represent DBD, while open rectangles and thick lines with letters stand for functional domains, which do not necessarily correspond to structural domains.

Cryptic structural domains were found in 53 human TFs (see Additional file 1). Although the 3D structures of cryptic structural domains remain undetermined and cannot be inferred by homology search methods, functional roles have been experimentally assigned to some of them. Figure 4 shows two examples of such cases.

A cryptic structural domain predicted in the MHC class II regulatory factor, RFX1 (conserved regions B, C, and D in Fig. 4a), has been found to mediate dimerization and transcriptional repression [40]. Human RFX1, 2, and 3 that bind DNA as homo- or hetero-dimeric complexes possess highly conserved B-C-D regions, to which the domain responsible for dimerization has been mapped [41,42].

The four cryptic structural domains at the C-terminus of the sterol regulatory element-binding protein 1a, SREBP-1a, correspond to the regulatory domain [43,44] as a whole (Fig. 4b). We cannot determine the exact number of structural domains, because the DICHOT system is not accurate enough to definitively delimit cryptic structural domains. SREBP-1a has two transmembrane helices in the middle (blue regions in Fig. 4b) and has both the N- and C-terminal regions in the cytoplasm. Upon binding of the C-terminal regulatory domain to SREBP-cleavage activating protein (SCAP), the N-terminal side of the protein is known to be cleaved off by specific proteases and be transported to the nucleus to act as a transcription activator [45].

All the results of the present study obtained by the DICHOT system can be retrieved at .

### Comparison with the previous study

Figure 4 also presents structural domains and ID regions assigned in the previous study (rectangles without black rims) (Minezaki et al. 2006). Notable differences between the present and previous assignments exist in p53 tumor suppressor (Fig. 4c). p53 has four functional regions, the acidic TAD at the N-terminus (TA), DBD, the oligomerization domain (Oligo), and the C-terminal negative regulatory domain (CNR). While DBD and Oligo were identified as structural domains as before, the present study departs from the previous one in assigning TA and CNR as ID regions. The finding of the present study is consistent with results of NMR experiments in which both TA and CNR fragments were revealed to be unstructured in free forms but become structured upon binding to partners: MDM2 in the case of TA [11,12,14] and S100B(bb) in the case of CNR [17]. Both complexes were co-crystallized and the 3D structures of the co-crystals were determined. The active form of p53 is a homotetramer resulting from dimerization of dimers [46] through the Oligo domain, which belongs to the all-α fold with four-helical bundles [46].

Hypoxia inducible factor 1α (HIF-1α) is composed of DBD (HLH), two PAS domains, an oxygen-dependent degradation domain (ODD), and two TADs termed N-TAD and C-TAD (Fig. 4d). Although C-TAD was considered to be a structural domain in the previous study because it becomes structured upon binding to the partner protein, an NMR study has revealed that the C-TAD fragment is unstructured in the unbound state [47,48]. Thus, the region should be classified as predicted in the present study. Similarly, N-TAD and ODD, both of which are unstructured unless bound to their partner proteins [49], should be classified as ID regions, as correctly done in this study.

## Discussion

A natural first step in analyzing the molecular architecture of proteins with ID regions is an accurate classification into structural domains and ID regions. For the dichotomic purpose, we have developed the DICHOT system, which combines existing methods of domain identification and disorder prediction with CLADIST, a newly developed classification program. The most reliable among the components is the identification methods that, along the sequence of a query protein, locate structural domains homologous to data compiled in SCOP and/or PDB. DICHOT identifies known domains by this method first and divides the remainder into structural domains and ID regions by a combination of the DISOPRED2 and CLADIST programs. The resolving power largely depends on the number of homologues available. The CLADIST program, in which the effect of sequence conservation is incorporated through local amino acid composition, maintains high accuracy even in cases in which only a limited number of homologues are available (Table 1).

The application of DICHOT to TFs has revealed that the fraction of structural domains is only 38% in human TFs, while that in *E. coli *TFs is as high as 95% (Fig. 3a). At the same time, DICHOT uncovered cryptic structural domains in 4% and 12% of the residues of human and *E. coli *TFs, respectively (Fig. 3a). We believe that the cryptic structural domains serve as good targets of structural genomics research. The smaller fraction of cryptic structural domains in human TFs than in *E. coli *TFs may reflect the more experimental research carried out on the former than the latter. Rapid increase of structural data in the PDB is expected to convert cryptic structural domains into known structural domains. In fact, besides RFX1 and SRBP-1a in Figure 3, transcription factor E2F1 was regarded as a good example of a structural domain whose structure had not been determined but with known function in the previous study [9], but the domain was assigned as a known structural domain in the present study, because the X-ray structure of the dimerization domain of E2F-1 had been solved in the meantime [50]. This conversion applies not only to E2F-1, but also to all members of the human E2F family[51]: E2F1-6 and DP1, 2. That the fraction of cryptic structural domains detected in this study was rather small indicates that the 3D structures already available include a high fraction of naturally occurring protein folds.

Cryptic structural domains were sometimes supported by the presence of Pfam domains in the same regions. However, we chose not to utilize Pfam for detection of structural domains, because some Pfam domains exist within ID regions [52,53]. For instance, the Pfam domain, PF02166, resides within the N-terminal side of the androgen receptor (Fig. 1), which is unstructured when not bound to the cognate protein as observed by spectroscopic measurements [13]. This happens presumably because a Pfam domain is defined for a sequence pattern conserved within a number of proteins, irrespective of the presence or absence of a globular structure [54,55]. It may apparently look contradictory that a Pfam domain is assigned to the N-terminal part of androgen receptor due to high sequence conservation on one hand, while on the other hand an ID region was assigned to the same region by our system due to high sequence divergence. However, there is no inconsistency because the N-terminal sequence is conserved only within homologues of phylogenetically close species as mentioned before, while the C-terminal sequence is conserved over more remote homologues (Fig. 1), and the poorer sequence conservation relative to the C-terminus is symptomatic of the existence of an ID region at the N-terminus.

Distinction of whether or not a globular structure is formed is crucial in the classification of the region into a structural domain or an ID region. Protein-protein interaction sites including TADs, located in the middle of long stretches of ID regions, are unstructured in the isolated state, even though they transiently adopt fixed configurations in complex with partner proteins. In this sense, they are regarded as intrinsically unstructured [4,11-17]. However, TADs have a sequence characteristic different from that of typical ID regions: they exhibit a significantly higher propensity to form a-helices and b-strands. Some attempts have been made to predict protein-protein interaction sites within ID regions based on this difference in sequence [56,57]. While identification of functional sites in ID regions is of biological importance, our concern in the present study was to divide protein molecules completely and accurately into structural domains and ID regions. Because of the intermediate nature of TADs between structural domains and ID regions, we paid special attention to distinguish structural domains and TADs: our system uses the criteria that TADs are shorter in length and are less conserved among homologues than structural domains. Even though we set the cut-off value large enough (50 residues) to cover all known TAD fragments, our method works well not only for TADs, but also for short SDs: many DBDs of less than 50 residues were correctly classified as SDs. As a result, the TADs erroneously assigned as structural domains in the previous study were reclassified as ID regions (Fig. 4).

## Conclusion

We developed the system DICHOT to completely divide proteins into structural and un-structural regions. The system includes structural assignments by homology searches, and the DISOPRED2 program as well as the new program CLADIST for ID region prediction. CLADIST takes un-aligned regions into consideration to enable DICHOT to divide entire amino acid sequences into structural/un-structural regions. The resulting classification of protein molecules was shown to be highly reliable. As a natural extension, we will apply the method to all human proteins. The research will provide an accurate ratio of structural domains to ID regions. Moreover, it is expected to uncover a number of cryptic structural domains in human proteins, which, because of the high degree of reliability, may become targets of structural study.

## Methods

### CLADIST, a in-house program for CLAssifying intrinsically DIsorder regions and STructural domains

We developed a program, CLADIST, for classifying intrinsically disorder regions and structural domains. CLADIST utilizes information of ID regions of a query sequence that cannot be BLAST-aligned with those in homologs even if structural domains are aligned. Figure 1 schematically shows a multiple alignment based on a BLAST search, where the solid and dotted lines represent the aligned and unaligned regions, respectively. We created multiple alignments based on segmental BLAST alignments in order to estimate local amino acid compositions. CLADIST uses the amino acid composition of the 21-residue window centered at each residue site of a query sequence. The local amino acid composition within the window was quantified taking all residues and gaps in the multiple alignments into account, with gaps treated as the 21st element. Here, we regard unaligned sites marked by dotted lines in Figure 1 as 'gaps' as well, in order to incorporate the information of unaligned regions. It follows that the local composition of a residue within a structural domain is expected to contain only a small number of gaps, i.e., a large number of similar sequences, whereas that of a residue within an ID region is likely to have a large number of gaps, i.e., a small number of similar sequences. After the above-described procedures, a 21-dimensional vector (20 dimensions for amino acids, one dimension for gaps) is assigned to each site in a query and is used for classification of the residue into structural domains and ID regions. The CLADIST program was equipped with support vector machines in the statistical package R .

We used the DisProt [39] database to train and test the CLADIST program. From DisProt, we selected 58 proteins that have at least one structural domain and one ID region longer than 30 residues, and that belong to the four representative eukaryotes: human, mouse, rat, and yeast. Structural domains were assigned by reverse PSI-BLAST searches against the PDB with the e-value cutoff of 1.0 × 10^-3^. The total numbers of sites in structural domains and ID regions are 3,746 and 1,497, respectively. All the sites were divided into 4 sections by generating random numbers. The training was conducted using 3 sections, with the remaining section utilized as the test data set. This procedure was repeated four times with different sets assigned as the test data set, and the average was calculated. For each query protein in the training data sets, BLAST searches were conducted against a genome database containing proteins from 621 organisms, with low-complexity regions in the database sequences and the query masked by SEG [58]. The redundancy of the genome sequences was reduced with a 90% identity cutoff. Aligned sequences with the e-values less than 1.0 × 10^-3 ^were accepted as homologues. Before quantification of amino acid composition, masked regions were replaced by the original sequences.

### The DICHOT system to divide proteins into structural domains and ID regions

The DICHOT system was constructed by combining three methods of structural domain identification, disorder prediction by DISOPRED2, and classification by the CLADIST program. Figure 2 shows a flow chart of the DICHOT system together with the results of homology searches and ID predictions illustrated in the upper half, together with the status boxes showing the assigned regions after each step.

In step 1, we conducted structural domain (SD) searches, i.e. BLAST and reverse PSI-BLAST searches against the PDB (Apr. 6, 2007 version) and SCOP [49] (version 1.69) and HMMER searches against SCOP. Because the assignments of structural domains above are conducted and stored in the GTOP database[59,60] in a genome-wide scale, we took analyzed data, including DISOPRED2 prediction [3], from GTOP. Trans-membrane (TM) regions were assigned according to the Swiss-Prot annotations, and the CLADIST program was run on all query sequences. The bar diagrams in the top-most four rows show the search results of a hypothetical protein.

In step 2, the DICHOT system accepts the search results in step 1 in the following descending order: the structural domain searches including TM, DISOPRED2, and CLADIST. For structural domain assignments based on SD searches, we specify the following conditions. The e-value cutoff of was set at 1.0 × 10^-3 ^except for the special cases described below, and if multiple hits of the same region were obtained, the best hit was chosen in the following descending order: BLAST against the PDB, reverse PSI-BLAST against the PDB, reverse PSI-BLAST against SCOP, and HMMER against SCOP. We chose this ordering because the profile methods such as PSI-BLAST and HMMER tend to provide elongated alignments resulting from over-assignment of regions flanking genuine ones. Although the e-value cutoff was generally set at 1.0 × 10^-3^, we found many HMMER hits with larger e-values to be true hits in the case of zinc-finger domains. Thus, we adopted a less stringent cutoff e-value of 1 for zinc fingers in HMMER searches.

The lengths of the structural regions selected by the above criteria were checked and the regions longer than or equal to 50 residues were accepted as known domains (red boxes). This length criterion was adopted by assessing the lengths of known TAD fragments. For the shorter regions, the SCOP classification of aligned domains was examined and those aligned to the SCOP structural domains of classes from 'a' (all-α), 'b' (all-β), 'c' (α/β), 'd' (α+β), 'e' (multidomain), 'f' (membrane), and 'g' (small), but not 'h' (coiled coil), 'i' (low resolution protein structure), and 'j' (peptide) were classified as known domains. As SCOP domains of classes h and j, as found in synuclein [61] and a p53 fragment [62] among others, do not adopt globular structures, they are classified as IDs. In the status box, the right green section of SD searches is assigned as a known domain, while the left one is left unclassified at this stage (hatched rectangle) because the domain is shorter than 50 residues and does not belong to any of the SCOP domains of classes a to g.

A structural domain predicted by CLADIST is not accepted if more than half of the region overlaps with structural domains assigned by SD searches. Out of the two structural domains predicted by CLADIST (yellowish green bars), the right one was neglected because a majority of the region overlaps with the structural domains assigned by SD searches. DICHOT admits all ID regions predicted by DISOPRED2 and CLADIST if they fall outside of structural domains assigned by SD searches. If ID regions predicted by DISOPRED2 overlap with the structural domains predicted by CLADIST, they are classified as ID regions. Due to interruption by an ID region, the left structural domain predicted by CLADIST (yellowish green bar) is divided into two enclaves (hatched boxes), both of which await classification in later steps.

In step 3, DICHOT examines the short structural domains left unclassified in step 2. A short structural domain found to coincide with a structural domain predicted by CLADIST is regarded as a known domain. Otherwise, it is classified as an ID region, as in the case of the right-most hatched rectangle in step 2. This additional requirement for short structural domain was introduced to prevent functional sites in ID regions from erroneously assigned as structural domains. For example, the 3D structures of TADs complexed with partner proteins have been determined and registered in the PDB. As they are classified as ID regions by the CLADIST program, they are correctly classified as ID regions by this step. Structural domains predicted by CLADIST whose lengths are longer than or equal to 30 amino acid residues are considered as candidates for cryptic domains. The middle hatched box in step 2 is shorter than 30 residues and therefore discarded at this step, while the left-most one is long enough to be left as a cryptic domain candidate.

In the fourth and final step, overlaps of cryptic domain candidates with known domains are checked. This step is necessary because some known domains arise at step 3 and they had not been subjected to the overlap check at step 2. If less than half of the regions overlap with any of the known domains, the regions are classified as cryptic domains (orange region). Any query sequence is thereby entirely classified into two categories, i.e., structural domains consisting of known and cryptic domains, and ID regions.

### Datasets

The dataset of 401 human TFs used in the present study is identical to that in the previous study [9]: data having direct experimental evidence in the Swiss-Prot database [63] (version 52.1) were collected, from which general transcription factors like TFIIB, TBP (TATA-box binding protein) and various co-acting factors involved in the transcription complex were removed. 229 *E. coli *TFs were taken from the GTOP-TF database  to constitute the reference set.

## Authors' contributions

SF developed the CLADIST program and the DICHOT system, and applied it to human TFs. SF also drafted the manuscript. KH assessed the results and participated in drafting the manuscript. YM provided the human TF dataset. TG supervised the research project. KN coordinated the study, assessed the results, and drafted the manuscript.

## Supplementary Material

Additional file 1 **List of proteins with cryptic domains**. The protein names, swiss-prot IDs, ENSEMBL IDs, ID regions, structural domain regions, and cryptic domain regions are listed. Click here for file
