# Supplementary material for: Development of an accurate classification system of proteins into structured and unstructured regions that uncovers novel structural domains: its application to human transcription factors
Source: BMC Struct Biol. 2009 Apr 30;9:26. doi: 10.1186/1472-6807-9-26 (PMC2687452; doi:10.1186/1472-6807-9-26)
Supplement: Additional file 1 — List of proteins with cryptic domains. The protein names, swiss-prot IDs, ENSEMBL IDs, ID regions, structural domain regions, and cryptic domain regions are listed. [file 1472-6807-9-26-S1.pdf]

Supplement Table S1: List of proteins with cryptic domains

family	name	Swiss-Prot	ENSEMBL	length	structural domains	ID regions	cryptic domain regions
AP2	AP-2 alpha	AP2A_HUMAN	ENSP00000259733	437	239	198	121-214,268-339,
	AP-2 beta	AP2B_HUMAN	ENSP00000342252	449	279	170	132-265,276-347,
	AP-2 gamma	AP2C_HUMAN	ENSP00000201031	450	250	200	165-269,281-345,
CTF/NF-1	NF1-A	NFIA_RAT	ENSP00000294608	504	184	320	1-184,
	NF1-B	NFIB_HUMAN	ENSP00000341192	420	185	235	1-185,
	NF1-C	NFIC_HUMAN	ENSP00000342859	508	182	326	1-182,
	NF1-X	NFIX_HUMAN	ENSP00000264826	501	183	318	1-183,
Homeobox	AT motif-binding factor	ATBF1_HUMAN	ENSP00000268489	3703	1213	2490	1280-1364,3009-3093,
Myb	A-Myb	MYBA_HUMAN	ENSP00000332150	744	243	501	501-592,
	B-myb	MYBB_HUMAN	ENSP00000217026	700	235	465	479-562,
	C-myb	MYB_HUMAN	ENSP00000340574	640	219	421	451-517,
	N-CoR1	NCOR1_HUMAN	ENSP00000268712	2440	265	2175	236-325,
	N-CoR2	NCOR2_HUMAN	ENSP00000348551	2514	456	2058	1314-1375,1604-1708,
PHD	Peregrin	BRPF1_HUMAN	ENSP00000306297	1214	434	780	340-455,
	HRX	HRX_HUMAN	ENSP00000352262	3969	724	3245	1872-2079,
RFX	RFX1	RFX1_HUMAN	ENSP00000254325	979	402	577	570-895,
	RFX2	RFX2_HUMAN	ENSP00000306335	723	389	334	335-406,420-593,604-672,
	RFX3	RFX3_HUMAN	ENSP00000265775	749	400	349	320-391,404-657,
TEA	TEF-1	TEAD1_HUMAN	ENSP00000354588	426	307	119	31-119,209-426,
	TEF-3	TEAD3_HUMAN	ENSP00000345772	434	308	126	30-119,217-434,
	TEF-4	TEAD4_HUMAN	ENSP00000352926	434	301	133	41-123,217-434,
bHLH	C-myc	MYC_HUMAN	ENSP00000259523	439	142	297	2-61,
	SREBP-1	SRBP1_HUMAN	ENSP00000261646	1147	569	578	567-818,864-985,1080-1146,
	SREBP-2	SRBP2_HUMAN	ENSP00000354476	1141	501	640	560-648,660-817,867-945,1076-1140,
	WS-bHLH	WBS14_HUMAN	ENSP00000320886	852	308	544	70-186,740-839,
bZIP	ATF-6A	ATF6A_HUMAN	ENSP00000263291	670	172	498	544-638,
	p45 NF-E2	NFE2_HUMAN	ENSP00000312436	373	147	226	290-370,
	NF-E2 related factor	NF2L1_HUMAN	ENSP00000323018	772	212	560	1-105,
bZIP-Maf	MafK	MAFK_HUMAN	ENSP00000344903	156	119	37	65-142,
f-C2H2/BTB/PO	Miz-1	ZBT17_HUMAN	ENSP00000235455	803	559	244	640-711,
	Zinc fingure protein 278	ZN278_HUMAN	ENSP00000266269	687	454	233	518-584,
	Zinc fingure protein 482	ZN482_HUMAN	ENSP00000335232	424	302	122	208-300,
zf_C2H2/KRAB	BRC1744	ZNF45_HUMAN	ENSP00000269973	682	642	40	74-142,
	eZNF	TCF17_HUMAN	ENSP00000337122	605	590	15	55-185,
	Zfp-37	ZFP37_HUMAN	ENSP00000259391	630	580	50	86-272,
	KOX4	ZNF7_HUMAN	ENSP00000320627	686	663	23	44-219,
	KOX27	ZNF43_HUMAN	ENSP00000352990	803	749	54	68-143,
	KOX31	ZN33A_HUMAN	ENSP00000304268	810	760	50	54-130,167-297,
	HPF4	ZNF85_HUMAN	ENSP00000329793	594	555	39	72-154,
			ENSP00000352919	516	513	3	1-70,
			ENSP00000347158	438	418	20	56-144,
zf-C2H2/etc	basonuclin-1	BNC1_HUMAN	ENSP00000307041	994	365	629	32-210,
	Ikaros	IKAR_HUMAN	ENSP00000352123	519	321	198	225-378,
	ZNF133	ZN133_HUMAN	ENSP00000246045	653	521	132	144-217,
	DB1	ZN161_HUMAN	ENSP00000258963	521	259	262	102-175,
	ZNF161	ZN161_HUMAN	ENSP00000258963	521	259	262	102-175,
	HF.10	ZNF35_HUMAN	ENSP00000296092	519	394	125	118-182,
zf-C4	NR2E1	NR2E1_HUMAN	ENSP00000230083	385	338	47	122-183,
	NR2E3	NR2E3_HUMAN	ENSP00000317199	409	338	71	152-218,
zf-C2HC	MyT1	MYT1_HUMAN	ENSP00000354922	1121	392	729	1023-1121,
zf-NF-X1	NF-X1	NFX1_HUMAN	ENSP00000263220	1070	648	422	360-572,
HMG box	SOX-5	SOX5_HUMAN	ENSP00000308927	763	132	631	160-226,
YL1	VPS72	VPS72_HUMAN	ENSP00000295315	364	63	301	167-229,
